# Investigating Age-Related Neural Compensation During Emotion Perception Using Electroencephalography

**DOI:** 10.3390/brainsci10020061

**Published:** 2020-01-23

**Authors:** Tao Yang, Caroline Di Bernardi Luft, Pei Sun, Joydeep Bhattacharya, Michael J. Banissy

**Affiliations:** 1 Department of Psychology, Tsinghua University, Beijing 100084, China; peisun@tsinghua.edu.cn; 2 Department of Psychology, Goldsmiths, University of London, London SE14 6NW, UK; j.bhattacharya@gold.ac.uk (J.B.); m.banissy@gold.ac.uk (M.J.B.); 3 School of Biological and Chemical Sciences, Queen Mary University of London, London E1 4NS, UK; luft@qmul.ac.uk

**Keywords:** emotion perception, ageing, neural compensation, electroencephalogram

## Abstract

Previous research suggests declines in emotion perception in older as compared to younger adults, but the underlying neural mechanisms remain unclear. Here, we address this by investigating how “face-age” and “face emotion intensity” affect both younger and older participants’ behavioural and neural responses using event-related potentials (ERPs). Sixteen young and fifteen older adults viewed and judged the emotion type of facial images with old or young face-age and with high- or low- emotion intensities while EEG was recorded. The ERP results revealed that young and older participants exhibited significant ERP differences in two neural clusters: the left frontal and centromedial regions (100–200 ms stimulus onset) and frontal region (250–900 ms) when perceiving neutral faces. Older participants also exhibited significantly higher ERPs within these two neural clusters during anger and happiness emotion perceptual tasks. However, while this pattern of activity supported neutral emotion processing, it was not sufficient to support the effective processing of facial expressions of anger and happiness as older adults showed reductions in performance when perceiving these emotions. These age-related changes are consistent with theoretical models of age-related changes in neurocognitive abilities and may reflect a general age-related cognitive neural compensation in older adults, rather than a specific emotion-processing neural compensation.

## 1. Introduction

The perception of emotional facial expression plays an important role in interpersonal communication [1,2]. Emotional expression can alter the meaning of speech and the ability to accurately identify emotional content is particularly important in social interactions [2]. Difficulties with emotion perception are associated with specific types of social impairment, including poor interpersonal interaction, reduced social competence and loneliness (e.g., [3,4]). These outcomes can have negative impacts on health and wellbeing (e.g., [5]), thus establishing how the capacity for emotion perception is affected as a function of normal adult aging is an important challenge.

Most studies have consistently demonstrated that older adults appear to have declined perception of some negative facial expressions of emotions, such as anger, sadness, fear and surprise (e.g., [6,7,8,9,10]). In contrast, age-related decline in the perception of happiness is less consistent: early studies suggested similar performance levels for young and older adults when using prototypical emotions [8,11,12] (see [1] for a review), but recent work suggests that there may be differences when more subtle emotional stimuli are used [13].

In addition, one issue involved in previous studies comparing both young and older people’s face perceptual abilities is the other-age effect—participants tend to show superior performance in the perception of own versus other age faces [14,15,16,17]. Therefore, the decline in the performance of older adults might be due to the use of young adult actors in the task, which favours young adult participants. It is also known that irrespective of the age of the perceiver, processing different age faces expressing the emotion can also influence emotion perception more broadly. For instance, in some studies, emotion perceptual accuracy of older faces was reported to be lower than young faces, as the wrinkles and folds of older faces can reduce the signal clarity of facial expressions [18,19,20]. Further, some researchers proposed that smile/happiness was easier to be perceived in young faces than older faces (e.g., [21]), whereas decoding facial expression of anger was higher in older faces than younger faces [22].

### Age-Related Neural Compensation in Emotion Perception

Functional neuroimaging studies have found that older participants exhibit a different neural activation pattern from younger participants during emotion perception [23,24,25,26]. Generally, older people tend to recruit more frontal cortical regions during emotion perception. For example, Gunning-Dixon, Gur, and Perkins [23] investigated age-related neural activations in cortical and limbic regions using functional magnetic resonance imaging (fMRI) by presenting facial stimuli of a mixture of negative emotions to both young and older participants. They observed neural activation in the amygdala and surrounding temporolimbic regions in younger participants, whereas older participants showed neural activations in left inferior and middle frontal regions. Similar results were found by Tessitore and colleagues [24] who compared older and younger participants’ neural processing of fearful and threatening stimuli using fMRI and found older participants were associated with increased prefrontal cortical neural responses and lower neural responses in the amygdala and posterior fusiform gyri relative to younger adults.

These studies have uncovered important age-related neural correlates of emotion processing; however, there are limitations. Firstly, most neuroimaging studies have typically investigated facial displays of only negative emotions [23,24,25,26]; therefore, it is not entirely clear whether the age-related compensation patterns reflect older people’s general emotion processing or processing of negative emotions only. Second, perceptual biases may have influenced previous neuroimaging results, since most have only used younger faces, which may lead to the other-age effect influencing performance [14,17]. Another set of issues that have largely been overlooked in most previous studies is the variation of emotional intensity or task difficulty. Previous studies have found that older adults have more difficulty than younger adults at labelling facial emotion with less intensity [27]. Further, recent work has suggested that age-related differences in emotion matching might be limited to low-intensity expressions [28]. However, high-intensity facial emotions displays that are typically linked with high performance have been used in most neuroimaging studies. This may potentially mask group differences in performance (e.g., see [13]), which leads to two issues: (1) it is unclear how older people’s perception of subtle/low-intensity facial expressions are linked with brain activity; and (2) it is unclear how task difficulty/face emotion intensity interacts with age-related neural activation changes.

In addition, much prior work using neuroimaging to investigate emotion processing in ageing has used fMRI [23,24,25,26,27,28,29,30,31,32,33,34,35,36]. Electroencephalography (EEG) has better temporal resolution than fMRI and enables inference about the neural time course of emotional facial expression processing. However, most previous ERP studies focused on studying young people’s ERPs during perceiving emotions (e.g., [20,29]). Those ERP studies that have compared young versus older neural activation differences mostly investigated neural activations within specific emotion-related ERP components that were previously defined by studying younger people (e.g., early posterior negativity (EPN)). For example, in one study, the late positive potentials (LPP) amplitudes were investigated as a function of age (between 18–81 years) and revealed that the LPP for negative images decreases linearly with age, but LPP for positive images is age-invariant across most of the adult life span [31]. Wieser et al. [32] compared young and older people’s early posterior negativity (EPN, an index of early emotion discrimination) during emotion (neutral, positive and negative) discrimination tasks. They found that EPN (168–232 ms) was delayed in older adults compared to younger adults, but a later component (232–296 ms) was unaffected. This study suggested an age-related delay of early visual emotion discrimination, but the delay does not seem to influence the discrimination of emotional stimuli. Tsolaki et al. [33] compared older and younger adults’ neural responses within face-selective ERP component N170 during viewings of facial images of anger and fear. They found that the N170 early component was modulated by the type of emotions and tends to have larger amplitudes in the elderly than the young during the viewing of negative stimuli (such as “anger” and “fear”). Although these studies have revealed important age-related neural activation differences during emotion processing, it is still not clear: (1) whether older people also recruit additional frontal neural regions during the perception of neutral emotion, and (2) what the effects of face emotion intensity and face-age are in modulating the neural compensation patterns.

With these in mind, the aim of this study is to compare older and young adults’ neural activation patterns and behavioral responses during the perception of both low- and high- emotional intensities of anger and happiness facial emotions. In particular, the present study seeks to compare older and young people’s compensation patterns at neutral (baseline), easy (high emotional intensities) and hard (low emotional intensities) conditions of anger and happiness emotion perception. By doing so, we seek to learn more about older adults’ neural compensation patterns in processing positive (happiness) and negative (anger) facial emotions with different task difficulties, which could speak to contemporary theoretical models. One model of interest is the compensation-related utilization of the neural circuits hypothesis (CRUNCH) model [37]. This model suggests that older people’s processing inefficiencies lead the ageing brain to recruit additional neural resources to perform at the level of younger adults: at lower levels of task demand, older adults exhibit a region-specific neural overactivation pattern but they can achieve similar or equivalent behavioural performance as younger adults (successful neural compensation); however, beyond a certain level of task demand, the older adult brain falls short of sufficient neural activation and their behavioural performance declines compared to younger people (neural compensation failure). Based on the predictions of the CRUNCH model, we expect to find that different task demands (face emotion images with low- and high- emotional intensities) might require different levels of neural compensation in older adults.

## 2. Materials and Methods

### 2.1. Participants

Thirty-one adult human volunteers participated in our study. Participants were divided into two groups: younger adults (*N* = 16, 12 female, mean ± SD of age: 24 ± 6 years), and older adults (*N* = 15, 12 female, mean ± SD of age: 69 ± 9 years). The study was conducted in the U.K., and all participants were native English speakers, with no known history of neurological problems, dyslexia or other language-related problems. They had normal or corrected-to-normal vision (self-reported). They gave written informed consent prior to beginning the experiment. The experimental protocol was approved by the local ethics committee of the Department of Psychology at Goldsmiths.

Level of education, premorbid intelligence (National Adult Reading Test, NART) [34], handedness, screening tests of working memory (digit span) [35], and alexithymia trait [36] were recorded for all participants. Further, we also administered the Mini-Mental State Examination (MMSE) as screening for possible dementia in the older adults group [38]; all participants had scores higher than 24, a usual cut-off limit for dementia [39].

### 2.2. Stimuli

**Figure 1:**
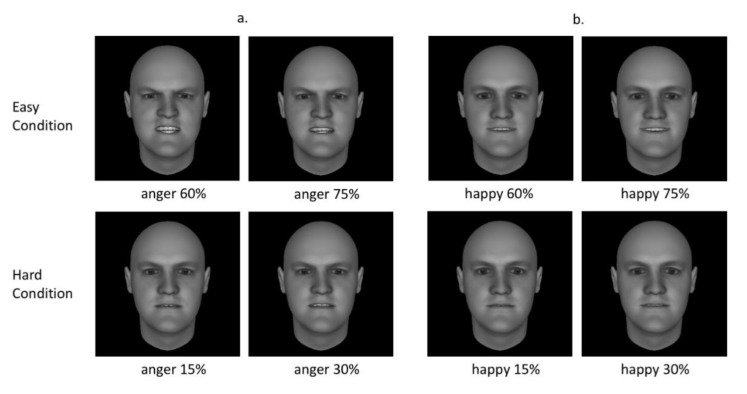
Example of emotional facial stimuli of anger (**a**) and happiness (**b**) used in the experiment. Hard task conditions contain facial stimuli with lower emotional intensities (15% and 30%), and easy task conditions contain facial stimuli with higher emotional intensities (60% and 75%).

The stimuli used in this study consisted of computer-generated human faces created by the FaceGen software ((www.facegen.com/products.htm), [40]), which were used in previous studies on emotion perception [18,41,42]. The facial stimuli had no hair or facial hair to avoid gender cues other than facial structure and features. All images were three-dimensional, greyscale, front profile Caucasian faces. Faces of thirty young (18–40 years; 15 female) and thirty old (65 years and above; 15 female) individuals were used. Each facial stimulus displayed anger or happiness in five emotional intensities: 0%, 15%, 30%, 60% and 75%. For both angry and happy faces, we formed two groups: faces with high intensities (60% and 75%), faces with low intensities (15% and 30%), corresponding to easy and difficult trials, respectively (see Figure 1 for examples). Faces with zero intensity correspond to neutral trials. Therefore, we had ten conditions in total (young/old face × angry/happy emotion × low/high intensity, and young/old face × neutral). Each condition comprised 60 experimental trials.

### 2.3. Experimental Procedure

**Figure 2:**
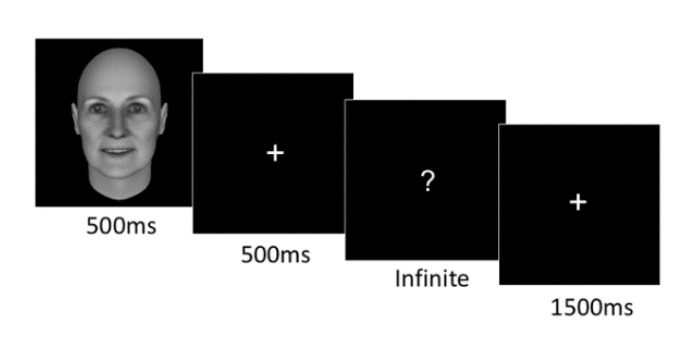
An outline of a trial. In each trial, a face was presented for 500 ms, followed by a blank screen with a fixation cross for 500 ms, and then followed by a prompt, asking participants to provide a response on the emotion category, happy or angry or neutral. The inter-trial interval was 1500 ms.

Participants were seated approximately 50 cm in front of a computer screen in a dimly lit, sound-attenuated room, and completed an emotion perception task. In each trial (Figure 2), a face was briefly presented for 500 ms, followed by a fixation cross for 500 ms, and then participants reported emotional category of the earlier face by pressing a key out of three options (anger, happiness, and neutral). The next trial started 1500 ms after the response (see Figure 2). At the beginning of the experiment, participants were informed that some facial expressions were subtle. There was a practice block (30 trials) before the experiment.

### 2.4. EEG Recording and Analysis

EEG signals were recorded with sixty-four Ag-AgCl electrodes placed according to the extended 10–20 electrode placement system, and amplified by a BioSemi ActiveTwo amplifier (www.biosemi.com). Four additional electrodes were placed above and below each eye, and at the outer canthus of each eye, to record vertical and horizontal eye movements, respectively. The sixty-four EEG signals were recorded with a sampling frequency of 512 Hz, bandpass filtered between 0.16–100 Hz. For the sixty-four EEG signals, MATLAB toolbox EEGLAB [43] was used for data preprocessing, and FieldTrip [44] for data analysis and statistical comparisons.

### 2.5. Preprocessing

The EEG data were algebraically re-referenced to a common average reference for ERP analyses because it is a less biased reference for comparing across scalp topographies [45,46]. The continuous data were high-pass filtered at 0.5 Hz and subsequently epoched from −1500 to 1500 ms time-locked to the onset of a face stimulus. The artifacts were treated in a semi-automated fashion. Visual inspection was initially made to remove any large artifacts, followed by an independent component analysis for correcting the eye blink-related artifacts. Subsequently, epochs containing amplitudes exceeding ±75 μV were discarded from future analysis. Each condition comprised of 60 trials; after artifact rejection, each condition had on average 42 trials remaining. The signals were further low-pass filtered at 35 Hz and epoched from −200 to 1000 ms following the stimulus presentation. The EEG signal of the epochs were averaged to obtain the ERP signals per condition. The ERPs were subsequently baseline corrected (−200 to 0 ms).

### 2.6. ERP Analysis

To identify the regions of interest (ROIs) on the Electrode x Time Space in the ERP differences between groups (old vs. young) while perceiving happy or angry emotion, we used a data-driven exploratory approach using a neutral emotion condition as a baseline. The ERPs of older and younger adults while perceiving neutral faces were compared by a non-parametric cluster-based permutation test [47], which is hypothesis-free, that controls the Type I error rate, and is widely used to analyse multidimensional EEG/MEG data [48,49,50]. The method consists of two steps as follows. First, the multidimensional (time, amplitude and electrode) clusters were detected by grouping neighboring data points that showed a significant effect (*p* < 0.05) of group (young vs. old) by independent *t*-tests, and a cluster-level statistic was subsequently calculated by summing the *t-*values in each cluster. Here, we considered electrodes with a distance of less than 5 cm as neighbors, yielding on average 4.2 neighbors per electrode. Second, Monte Carlo randomisation was used to calculate the exact probability that a cluster with the maximum cluster-level statistic was observed under the assumption that the ERP values of the two compared groups or conditions were not significantly different. A histogram of maximum cluster-level statistics was obtained by calculating the cluster-level statistic by repeating the entire analysis a large number of times (=500 in the current study) on random permutation of the pooled data of the two groups or conditions; this histogram was subsequently used to calculate the exact *p*-value for that cluster, and these procedures were subsequently carried out for the lower ranking cluster-level statistics [48]. The clusters thus obtained were subsequently used as the ROIs to find the main differences between the younger and older adults by standard factorial mixed ANOVA on the mean ERP amplitudes at these ROIs. By choosing a neutral condition as the baseline, this method can show the role of emotion type, task difficulty and face-age in modulating the same spatiotemporal brain profiles. Specifically, it can help revealing the participants’ neural changes from baseline to easy and hard emotion conditions, which provides the evidence for validating the age-related neural compensation hypothesis.

## 3. Results

### 3.1. Participants’ Characteristics

Demographic characteristics of the two groups are listed in Table 1. The two groups did not significantly differ (*p* > 0.05) in level of education, premorbid intelligence (NART), handedness, working memory (digit span), or Toronto Alexithymia Scale (TAS-20).

### 3.2. Behavioral Results

Prior to data analysis, accuracy outliers were excluded from further analysis (using a criteria of >3 standard deviations from the mean on any individual variable of interest and significance using Grubb’s test). Specifically, two datasets from *older face_neutral* condition, one dataset from *older face_anger_easy* condition, one dataset from *younger face_neutral* condition, one dataset from *younger face_anger_easy* condition, and two datasets from *younger face_happy_easy* condition were identified as outliers and excluded from further analysis. No RT outliers were found. Participants’ perceptual performance (accuracy and RTs) on each task were analysed.

#### 3.2.1. Perception of Neutral Facial Expression

**Figure 3:**
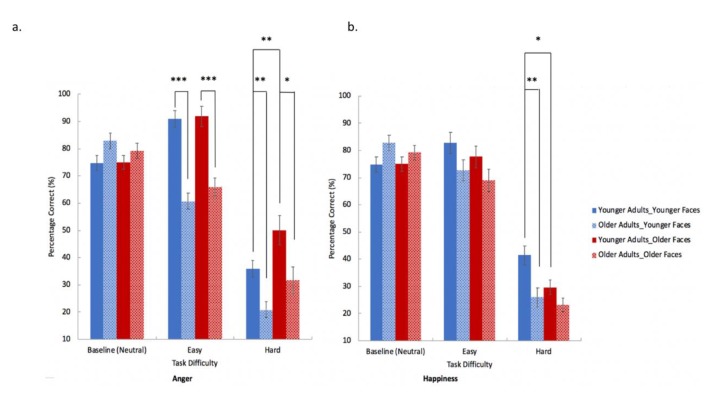
Older (patterned bars) and younger (solid bars) participants’ perceptual performance (accuracy) in anger (**a**) and happiness (**b**) experimental trials at different task difficulties (neutral (baseline), easy and hard). Bars in red represent trials with older face stimuli, bars in blue represent trials with younger face stimuli. (1) Anger perception (**a**): older group performed worse than younger group in both easy (old face stimuli condition and young face stimuli condition) and hard (old face stimuli condition and young face stimuli condition) levels. (2) Happiness perception (**b**): older adults performed poorer in hard condition using younger face stimuli, but not in other conditions. (3) In addition, within-group comparison revealed that younger participants’ performance on young and old face stimuli were significantly different for only hard level of anger and happiness perception tasks, with superior performance on younger face stimuli in happiness perceptual tasks and superior performance on older face stimuli in anger perceptual tasks. Error bars represents S.E. (*** represents *p* < 0.001. ** represents *p* < 0.01. * represents *p* < 0.05).

Perceptual performance on neutral emotion condition was analysed by a 2 × 2 mixed factorial ANOVA with group (young, old) as a between-participants factor and face-age (young, old) as a within-participants factor. Older adults showed marginally higher accuracy on recognizing neutral emotion than younger adults (*F*(1, 26) = 4.195, *p* = 0.051, *η ^2^*= 0.139; Figure 3). No other significant main effect or interaction was found (see Supplementary Material).

#### 3.2.2. Perception of Anger and Happiness Facial Expressions

Perceptual performance on anger and happiness trials were analysed by a 2 × 2 × 2 × 2 mixed ANOVA with group (young, old) as the between-participants factor, and emotion type (anger, happiness), face-age (young, old), and task difficulty (easy, hard) as within-participants factors. Younger adults showed overall better performance than the older adults (*F*(1, 26) = 30.357, *p* < 0.001, *η*^2^ = 0.539; Figure 3). The main effect of task difficulty was also significant (*F*(1, 26) = 381.512, *p* < 0.001, *η*^2^ = 0.936), which was due to the overall accuracy for easy trials being significantly higher than hard trials. The main effects of emotion type and face-age were not significant. The interaction of emotion type × face-age was significant (*F*(1, 26) = 24.495, *p* < 0.001, *η^2^* = 0.485). Pairwise comparisons (with Bonferroni correction) revealed that this was due to the overall accuracy for older face stimuli being significantly higher than younger face stimuli for anger emotion (anger: *p* < 0.001; *d* = 1.699). For the happy facial emotion, the overall accuracy for younger face stimuli was significantly higher than for older face stimuli (happiness: *p* = 0.008, *d* = 1.441, Bonferroni corrected).

The interaction of emotion type × face-age × task difficulty was also significant (*F*(1, 26) = 15.085, *p* = 0.001, *η^2^* = 0.367). Pairwise comparison (Bonferroni-corrected) revealed that participants’ overall accuracy for older face stimuli was significantly higher than younger face stimuli in the hard level of anger perceptual tasks (*p* < 0.001, *d* = 1.744). In the hard level of happiness perceptual tasks, participants’ overall accuracy for younger face stimuli was significantly higher than older face stimuli (*p* < 0.001, *d* = 1.538).

The interaction of emotion type × face-age × task difficulty × group was also significant (*F*(1, 26) = 4.893, *p* = 0.036, *η^2^* = 0.158). Pairwise comparison (Bonferroni-corrected) revealed that the older group performed worse than the younger group at anger perception in both easy (old face stimuli condition: *p* < 0.001, *d* = 1.608; young face stimuli condition: *p* < 0.001, *d* = 1.865) and hard (old face stimuli condition: *p* = 0.032, *d* = 1.203; young face stimuli condition: *p* = 0.008, *d* = 1.463) levels (Figure 3a). Older adults also performed poorer in hard condition of happiness perception using younger face stimuli (*p* = 0.008, *d* = 1.443), but not in other conditions (Figure 3b). In addition, within-group comparison revealed that younger participants’ performance on young and old face stimuli were significantly different for only the hard level of anger and happiness perception tasks (anger: *p* = 0.008, *d* = 1.438; happiness: *p* < 0.001, *d* = 1.512), with superior performance on younger face stimuli in happiness perceptual tasks and superior performance on older face stimuli in anger perceptual tasks. In contrast, older participants’ performances on younger and older face stimuli were not significantly different in both easy and hard levels of anger and happiness perception tasks.

### 3.3. ERP Analysis

#### 3.3.1. Selections of ERP Clusters that Showed Difference between Old and Young Group

**Figure 4:**
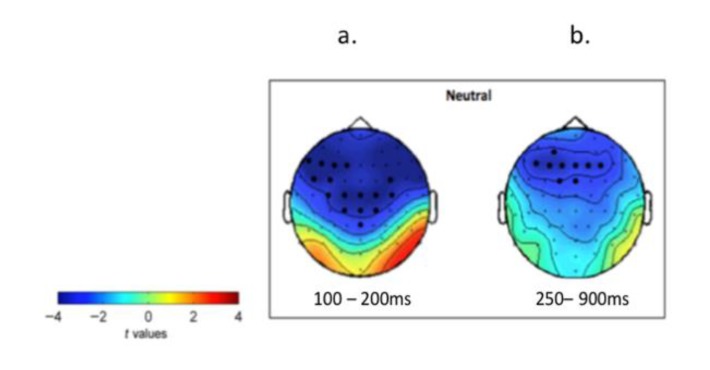
Significant clusters of the nonparametric cluster randomisation test comparing the two groups (young minus old) on neutral condition. First cluster time window: 100–200 ms, left-frontal and centromedial area (F1, F3, F5, F7, Fc5, Fc5, C1, C3, Cp1, Pz, Cpz, Fc4, Cz, C2, C4); second cluster time window: 250–900 ms; frontal area (AF3 F1, F3, F5, Fc1, Fz, F2, F4, Fcz).

In order to select the ROIs, we compared young and older participants’ ERPs (young vs. old) on neutral emotion condition by using the non-parametric cluster permutation tests (ERPs corresponding to the old and young faces stimuli trials of neural condition were merged). The significant clusters are shown in Figure 4. We identified two significant clusters: the first cluster was a left-frontal and centromedial cluster between 100–200 ms (Figure 4a), and the second cluster was over the frontal region between 250–900 ms (Figure 4b). These two spatiotemporally distinct clusters served as our two ROIs.

#### 3.3.2. The Effect of Emotion, Face-age and Task Difficulty in Modulating ERPs

For each cluster, we computed individual participant mean ERP amplitudes, and the data were subsequently analyzed by a mixed 2 × 2 × 2 × 2 ANOVA with emotions (anger, happiness), face-age (young and old), task difficulty (easy, hard) as within-participants factors, and group (young, old) as a between-participants factor.

***Cluster one***

**Figure 5:**
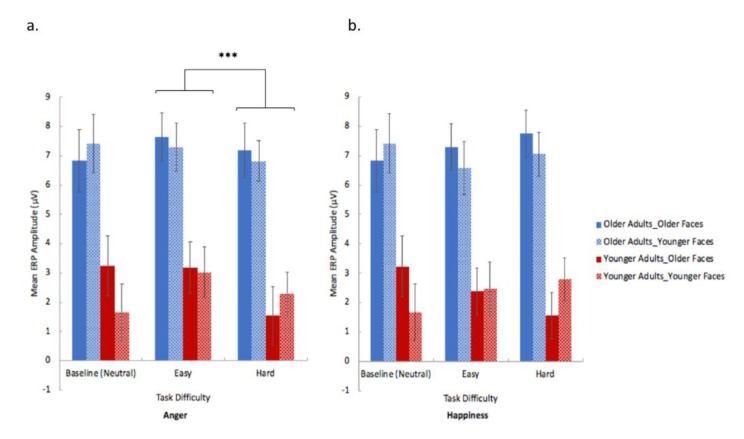
Older (bars in blue) and younger (bars in red) participants’ cluster one mean ERP amplitude for anger (**a**) and happiness (**b**) experimental trials at different task difficulties (neutral (baseline), easy and hard). Solid bars represent trials with older face stimuli; patterned bars represent trials with younger face stimuli. The overall left-frontal and centromedial mean ERP amplitude (100–200 ms) of easy condition was significantly higher than the hard condition in anger task, regardless of group. Error bars represents S.E. (*** represents *p* < 0.001).

The results revealed that older participants exhibited significantly higher overall mean ERP amplitudes than younger group (main effect of group, *F*(1, 26) = 20.273, *p* < 0.001, *η^2^* = 0.438; Figure 5). In addition, the easy condition (high-intensity facial expressions) elicited significantly higher overall mean ERP amplitudes in left frontal and centromedial regions (100–200 ms) than the hard condition (low-intensity facial expression) [the main effect of task difficulty, *F*(1, 26) = 4.974, *p* = 0.035, *η^2^* = 0.161]. The main effects of emotion type and face-age were not significant.

The interaction of face-age × group was significant [*F*(1, 26) = 5.775, *p* = 0.024, *η^2^* = 0.182]. Pairwise comparison (with Bonferroni correction) revealed that older participants exhibited significantly higher mean ERP amplitudes in left frontal and centromedial regions (100–200 ms) than younger participants in both older and younger face stimuli conditions (older face stimuli, *p* < 0.001, *d* = 1.764; younger face stimuli, *p* < 0.001, *d* = 1.583). No significant results was found in within-group comparisons (old group: *p* = 0.054 and young group: *p* = 0.172; with Bonferroni correction; Figure 5). The interaction of emotion type × difficulty was significant (*F*(1, 26) = 6.632, *p* = 0.017, *η^2^* = 0.201). Pairwise comparison revealed that the mean left-frontal and centromedial ERP amplitude (100–200 ms) of the easy condition was significantly higher than the hard condition in anger task (*p* < 0.001, *d* = 1.537) (Figure 5).

***Cluster two***

**Figure 6:**
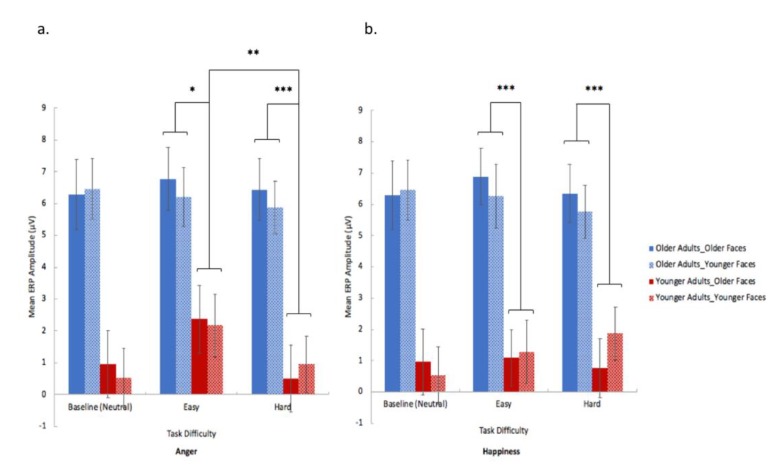
Older (bars in blue) and younger (bars in red) participants’ cluster two mean ERP amplitude for anger (**a**) and happiness (**b**) experimental trials at different task difficulties (neutral (baseline), easy and hard). Solid bars represent trials with older face stimuli; patterned bars represent trials with younger face stimuli. (1) Older participants exhibited significantly higher frontal ERP amplitudes (250–900 ms) in both easy and hard levels of anger and happiness perceptual tasks compared to younger participants. (2) Within-group comparison revealed that younger participants’ mean frontal ERP amplitudes (250–900 ms) for the easy condition was significantly higher than the hard condition in anger perception, but not significantly different in the happiness perceptual tasks; whereas older participants did not show significant different ERPs across easy and hard task conditions in both the happiness and anger perceptual tasks. Error bars represents S.E. (significances are marked by *** *p* < 0.001, ** *p* < 0.01, and * *p* < 0.05).

A 2 (emotion type) × 2 (task difficulty) × 2 (face-age) × 2 (group) mixed ANOVA on the ERP amplitudes of the second cluster (250–900 ms) revealed significant main effect of group (*F*(1, 26) = 17.051, *p* < 0.001, *η^2^* = 0.396), which was due to older participants exhibiting significantly higher overall mean ERP amplitudes than younger group (Figure 6). The main effect of task difficulty was significant (*F*(1, 26) = 6.813, *p* = 0.015, *η^2^* = 0.208), which was due to the easy condition (high-intensity facial expressions) eliciting significantly higher overall mean ERP amplitudes in frontal regions (250–900 ms) than the hard condition (low-intensity facial expression) (Figure 6). The main effects of emotion type and face-age were not significant.

The interaction of face-age × group was significant, *F*(1, 26) = 5.468, *p* = 0.027, *η^2^* = 0.174, which was due to older adults exhibiting significantly higher ERPs than younger adults in both older (*p* < 0.001, *d* = 1.641) and younger (*p* = 0.008, *d* = 1.428, Bonferroni corrected) face stimuli (Figure 6). In addition, within-group comparison revealed that the older group’s mean ERP amplitude for younger faces was significantly lower than for older faces (*p* = 0.047, *d* = 0.216), whereas the younger group’s mean ERP for young and old face stimuli did not differ *(p* = 0.221).

The interaction of emotion type × task difficulty × group was significant (*F*(1, 26) = 5.781, *p* = 0.024, *η^2^* = 0.182). Pairwise comparison (with Bonferroni correction) revealed that older participants exhibited significantly higher frontal ERP amplitudes (250–900 ms) in both easy and hard levels of anger (easy: *p* = 0.048, *d* = 1.367; hard: *p* < 0.001, *d* = 1.766) and happiness (easy: *p* < 0.001, *d* = 1.540; hard: *p* < 0.001, *d* = 1.511) perceptual tasks. In addition, the within-group comparison revealed that younger participants’ mean frontal ERP amplitudes (250–900 ms) for the easy condition was significantly higher than the hard condition in anger perception (*p* = 0.008, *d* = 1.487), but not significantly different in the happiness perceptual tasks, whereas older participants did not show significant different ERPs across easy and hard task conditions in both the happiness and anger perceptual tasks (Figure 6).

## 4. Discussion

The aim of the present study is to examine age-related changes in emotion perception and to further reveal the underlying neural mechanisms. Our study contributes to the current literature in two main ways. First, most neuroimaging studies have typically investigated people’s emotion perception by using facial displays of (1) only negative emotions, (2) only young faces, or (3) only high-emotional intensity. To overcome these limitations, we used an integrative approach by considering how “emotion type”, “face-age” and “face emotion intensity” affect younger and older participants’ emotion perception, all in the same study. Second, one of the main strengths and novelty of our study is related to our data-driven approach. We used an entirely data-driven approach to explore the age-related neural differences during emotion perception—this is an important contribution as we did not limit ourselves to the known ERP components (e.g., [29,30]). Our study provides additional insights by showing that: (1) older participants exhibited neural differences in the left frontal and centromedial region (100–200 ms stimuli onset) and frontal region (250–900 ms stimuli onset) at neutral condition, which suggests that older people’s neural compensation may start at the neutral (baseline) condition; (2) within the same neural clusters, older participants also exhibited similar patterns of neural activity during anger and happiness emotion perceptual tasks; (3) older people’s compensation was successful in processing neutral emotion as they exhibited similar behavioural performance as younger participants; however, this was not enough for them to successfully apply the compensation strategy in processing facial expressions of anger and happiness (hard condition). We now discuss these in more detail below.

### 4.1. Age-Related Behavioural Perceptual Performance Differences

For the perception of anger, older participants’ mean accuracy was significantly lower than younger participants in both hard and easy conditions. This finding suggests that older participants show declined abilities in processing both low- and high-intensities of anger facial expressions. This finding adds confirmation to prior work indicating that older participants show declined perception of anger (see [1] for review). For the perception of happiness, older participants showed poorer performance than younger participants in perceiving low-intensity/subtle happiness from younger faces; however, their perception of high-intensity happiness remain intact. This is consistent with previous findings that older participants exhibit significantly poorer performance in perceiving low-intensity happiness from older faces (e.g., [13]), but relatively intact performance on prototypical emotions displayed by younger adults [7,8,9,51]. Furthermore, it has recently been found that older adults are associated with larger congruency sequence effect [52,53] in the Stroop task compared to younger adult controls [54]. In future studies, it will be interesting to investigate the trial-to-trial carryover effect in emotion perception and to see how typical aging affects this interference effect.

### 4.2. Effect of Face-Age on Behavioural Perceptual Performance

For anger, younger participants’ accuracy for perceiving anger from old face stimuli was significantly higher than from younger faces in the hard condition, which is consistent with Hühnel et al.’s [22] finding that facial expression of anger is easier to detect from older faces than from young faces. This might suggest that some facial features (e.g., winkles around eyes and mouths) of older faces may exaggerate subtle expressions of anger. However, the superior performance in perceiving subtle anger from older faces was not found in older participants, as their performance on younger and older face stimuli was not significantly different in easy or hard levels of anger. It will be important to explore this question further in future work using alternative stimuli to those used in the current design.

For happy emotion, within-group comparison revealed that the younger participants’ performance in perceiving low-intensity happiness from younger faces was significantly better than from older faces, which is consistent with Murphy et al.’s finding [21] that smile/happiness is easier to perceive in young than older faces, whereas, older participants’ performance in perceiving happiness from younger and older faces did not significantly differ in either low or high intensity. Younger participants’ superior performance on younger faces trials (in low emotion intensity condition) might reflect “own-age bias” where people are better at perceiving faces of their own ages [55]. However, this “own-age bias” was not shown in older participants. Younger participants’ superior performance in perceiving subtle happiness from younger faces might partially account for the perceptual performance gap between younger and older participants. In other words, the reported poorer performance of older participants might not be entirely due to their perceptual decline, but younger participants’ superior perception of their own-age faces.

### 4.3. Facial Emotion Perception and Neural Compensation in Older People Revealed by ERPs

Previous ERP studies have revealed three major facial emotion-related components, which are early frontocentral positivity (around 120 ms stimulus onset), a later broadly distributed sustained positivity beyond 250 ms post-stimulus, and enhanced negativity at lateral posterior sites (EPN) [30,56,57]. The results of the current study revealed that older participants showed significantly higher early frontal and centromedial ERP positivity (100–200 ms) and a later sustained positivity beyond 250 ms post-stimulus across all facial emotions types including neutral emotion.

Based on earlier studies of Eimer et al. [29,30], the early and late frontal and centromedial positivity can be interpreted to reflect a non-automatic and attentive approach of emotion processing, which do not appear to be modulated by emotion type. Older people’s significantly higher early (100–200 ms) and late (250–900 ms) frontal and centromedial positivities compared to younger participants during emotion processing may be interpreted as evidence to suggest that older adults require more cortical neural resources than younger people in all types of emotion perception. This additional recruitment of frontal regions during processing facial emotional expression is in parallel with previous fMRI studies, which also found that older people showed significantly higher frontal neural activations compared to younger adults during emotion perception tasks (e.g., [24,25]).

One aim of the present study is to examine the relationship between emotion task difficulty (emotional intensity) and older participants’ age-related neural compensation patterns. The ERP results revealed that older participants exhibited significantly higher activations in the left frontal and centromedial regions (100–200 ms stimuli onset) and frontal region (250–900 ms stimuli onset) compared to young participants when perceiving neutral facial expression. This might suggest that neural differences between young and old participants may start at the neutral (baseline) condition in order to compensate for cortical processing inefficiency. In addition, older participants also exhibited similar patterns during perceiving easy- and hard- levels of anger and happiness facial expressions. When combined with the behavioural results, the findings seem to suggest efficient processing of neutral emotion as they performed equally well as younger participants on neutral emotion perceptual trials. A similar level of neural resources was not enough for older adults to successfully process facial expressions of anger (both easy and hard conditions) and happiness (hard condition), where they showed significantly poorer perceptual performance on both happiness (only hard condition) and anger (both easy and hard conditions) perceptual trials. This pattern of results seems in line with the CRUNCH model [37], which proposes that at a lower level of task demand, older participants exhibit a region-specific neural overactivation pattern, but they can achieve similar or equivalent behavioural as younger participants (successful neural compensation). However, beyond a certain level of task demand, there is insufficient neural activation and their behavioural performance declines compared to the young people (neural compensation failure).

It should be noted that the neural overactivation in left frontal and central regions (100–200 ms stimuli onset) and frontal region (250–900 ms stimuli onset) might not reflect emotion-specific neural compensation. Other aging studies have also shown that older people tend to recruit more frontal cortical regions than younger people when performing identical cognitive tasks, especially effortful tasks [58,59]. This has been found in other brain imaging studies, including melodic expectancy processing [60], attention [61,62,63], working memory [64,65] and executive functioning [66]. Therefore, the neural overactivation in frontal-centro regions might reflect a top-down and controlled neural compensation to suppress or inhibit irrelevant information rather than emotion-specific neural compensation (e.g., [67]). It is also important to note that we only compared the ERP amplitudes of the same clusters (young–old neural activation difference extracted from neutral condition), which might potentially ignore some emotion-specific neural compensation patterns.

### 4.4. Effects of “Emotion Type”, “Task Difficulty” and “Face-Age” on ERPs

The results showed that the effect of emotion type (happiness and anger) did not significantly modulate younger and older participants’ early (100–200 ms) or late (250–900 ms) positivities during facial emotion perceptual tasks. These findings are in line with previous studies that claimed the early and late positivities are not modulated by emotion type [29,30]. The effect of face-age seems to modulate older people’s frontal region ERP between 250–900 ms. Older group’s mean ERP amplitude for older faces was significantly higher than for younger faces, which might be due to that the facial expressions portrayed by older faces are harder to be processed compared to those portrayed by younger faces [18,19,20], whereas younger group mean ERP for young and old face stimuli did not differ, which might suggest that younger people’s neural processing for young and older emotional faces is similar and they need to recruit more neural regions to process older emotional faces. This finding is novel and awaits further replication.

In contrast, the effect of task difficulty (or facial emotional intensities) significantly modulated the overall ERP amplitudes of both early (100–200 ms) frontal and centromedial and late (250–900 ms) frontal brain regions in anger tasks, but not in happiness tasks. Specifically, the overall mean left-frontal and centromedial ERP amplitudes (100–200 ms) of easy condition (high emotion intensity) was significantly higher than hard condition (low emotion intensity) in anger task, regardless of group and face-age. In other words, higher intensities of anger elicited significantly higher early (100–200 ms) frontal and centromedial positivities in participants, regardless of group and face-age. Furthermore, younger participants’ mean frontal ERP amplitudes (250–900 ms) for the easy condition was significantly higher than the hard condition (low emotion intensity) in anger perception, but this pattern was not shown in the happiness condition. Whereas, older participants did not show significant different ERPs across easy and hard conditions in both happiness and anger perceptual tasks. In other words, higher intensities of anger elicited significantly higher late (250–900 ms) frontal positivities in younger participants only. These findings suggest that higher intensities of anger can trigger higher early (100–200 ms) frontal and centromedial positivities and late (250–900 ms, only in younger participants) frontal positivities. These findings seem consistent with Eimer et al.’s [29,30] interpretation that these two positivity components may contribute to attention-regulated emotional processing, as higher intensities of anger signal potential threat and they are normally associated with a higher level devotion of attention [68]. In addition, a recent study has also shown that anger elicits significantly higher late P3 component (350–450 ms at temporo-occipital and parieto-occipital channel locations) compared to happiness in both younger and older adults [69]. The present finding also partially agrees with a previous proposal suggesting high intensities of emotional images elicited larger late positive potentials than lower intensities of emotional images [70], as it is not the case for happiness perception in the present study.

### 4.5. Limitations and Future Avenues

Most of the emotion-related ERP components were conceptualized by observing younger adults’ ERPs (e.g., [29,30]). Later EEG studies that investigated older people’s emotion processing only compared young–old neural differences within these previously defined electrodes and time windows (e.g., [32,33]). However, ageing can lead to older people recruiting additional neural regions during emotion processing (neural compensation), that may extend beyond these already defined EEG neural sites. Recently, some ageing studies started to investigate how ageing affects the previously defined emotion-related components [31,32,33], but fewer studies have investigated how ageing and other factors (task difficulty, emotion type) affect the ERPs of other neural sites during perceiving facial emotions. In this study, instead of focusing on specific ERP components a priori, we adopted a data-driven approach and selected the ROIs based on the differences between younger and older adults for the neutral condition; therefore, the neutral condition serves as a baseline and helps to reveal the changes in participants’ neural responses from baseline to easy and hard emotion conditions. By using the neutral condition, we can examine the effects of emotion type and task difficulty in modulating participants’ age-related neural compensation patterns when processing facial expressions, which provides the evidence to demonstrate the age-related neural compensation. There is some evidence that even neutral is processed as an expression [71,72], therefore contrasting the ERP responses between young and older participants on neutral condition gave us the signal differences related to age when trying to recognize emotion in general. In addition, contrasting young and old neural responses to neutral faces enabled us to compare their responses to emotional faces which makes the analyses non-circular to avoid the danger of double-dipping as we tested the differences between the groups on data that we did not use to explore the age-related differences [73]. This is our second reason—as by doing this, we were able to use a data-driven approach to explore the data as a first step (using appropriate methods taking into account the multiple comparison problem) and to test these differences, an unseen dataset when stronger emotions of high and low intensity were being presented.

While useful to address this question, our approach might have ignored the group ERP differences in response to specific emotions, as prior studies have suggested that neutral, anger and happiness facial emotions are processed by different neural regions (e.g., [74,75]). In addition, our selections might have neglected emotion-specific neural compensation in other brain areas, which awaits further neuroimaging studies (e.g., fMRI) to investigate in depth.

## Figures and Tables

**Table 1:** Basic demographic and descriptive characteristics of the two study groups.

	*Old (n = 15)*	*Young (n = 16)*
**Gender (male/female)**	3/12	4/12
**Age (years)**	69.20 (8.809)	24.19 (5.576)
**Education (years)**	15 (3)	16 (2)
**Handedness (right/left)**	14/1	15/1
**Premorbid IQ (NART)**	120.07 (8.430)	115.44 (10.295)
**Working memory (digit-span)**	105.27 (16.867)	106.63 (12.209)
**TAS-20 score**	43.00 (7.329)	44.75 (9.692)

## Supplementary Materials

The following are available online at https://www.mdpi.com/2076-3425/10/2/61/s1. It include other results and findings that are not included in the main contents [results of RTs, results of accuracy and ERP analysis, results of ERP components P100 and P300].
