# Supplementary material for: Investigating Age-Related Neural Compensation During Emotion Perception Using Electroencephalography
Source: Brain Sci. 2020 Jan 23;10(2):61. doi: 10.3390/brainsci10020061 (PMC7071462; doi:10.3390/brainsci10020061)
Supplement: Supplementary file 1 [file brainsci-10-00061-s001.zip › supplementary files/Supplementary 2_data analysis.docx]

**Accuracy**

*Supplementary Table 1: Results of ANOVA identifying the effects of emotion type, face age, task difficulty and group (young vs. old) on accuracy of neutral emotion task.*

|  | *df* | *F* | *η²* | *p* |
| --- | --- | --- | --- | --- |
| Face Age | 1 | .681 | .026 | .417 |
| Face Age × Group | 1 | 1.121 | .041 | .300 |
| Error (Face Age) | 26 |  |  |  |
| Group | 1 | 4.195 | .139 | .051 |
| Error | 26 |  |  |  |

* *p* < .05, *** *p* < .001

*Supplementary Table 2: Results of ANOVA identifying the effects of emotion type, face age, task difficulty and group (young vs. old) on accuracy of happiness and anger emotion tasks.*

|  | *df* | *F* | *η²* | *p* |
| --- | --- | --- | --- | --- |
| Emotion Type | 1 | 1.302 | .048 | .264 |
| Emotion Type × Group | 1 | 1.755 | .063 | .197 |
| Error (Emotion Type) | 26 |  |  |  |
| Face Age | 1 | 2.021 | .072 | .167 |
| Face Age × Group | 1 | 1.192 | .044 | .285 |
| Error (Face Age) | 26 |  |  |  |
| Task Difficulty | 1 | 381.512 | .936 | < .001*** |
| Task Difficulty Group | 1 | .783 | .029 | .384 |
| Error (Task Difficulty) | 26 |  |  |  |
| Emotion Type × Face Age | 1 | 24.495 | .485 | < .001*** |
| Emotion Type × Face Age × Group | 1 | 1.582 | .057 | .220 |
| Error (Emotion Type × Face Age) | 26 |  |  |  |
| Emotion Type × Task Difficulty | 1 | 2.453 | .086 | .129 |
| Emotion Type × Task Difficulty × Group | 1 | .542 | .020 | .468 |
| Error (Emotion Type × Task Difficulty) | 26 |  |  |  |
| Face Age × Task Difficulty | 1 | 3.999 | .133 | .056 |
| Face Age × Task Difficulty × Group | 1 | .037 | .001 | .849 |
| Error (Face Age × Task Difficulty) | 26 |  |  |  |
| Emotion Type × Face Age × Task Difficulty | 1 | 15.085 | .367 | .001* |
| Emotion Type × Face Age × Task Difficulty × Group | 1 | 4.893 | .158 | .036* |
| Error (Emotion Type × Face Age × Task Difficulty) | 26 |  |  |  |
| Group | 1 | 30.357 | .539 | < .001*** |
| Error | 26 |  |  |  |

* *p* < .05, *** *p* < .001

**Reaction Times (RTs)**

*Supplementary Table 3: Results of ANOVA identifying the effects of emotion type, face age, task difficulty and group (young vs. old) on RTs of neutral emotion task.*

|  | df | F | *η²* | p |
| --- | --- | --- | --- | --- |
| Face Age | 1 | .093 | .003 | .763 |
| Face Age × Group | 1 | .215 | .007 | .647 |
| Error (Face Age) | 29 |  |  |  |
| Group | 1 | .500 | .017 | .485 |
| Error | 29 |  |  |  |

* *p* < .05, *** *p* < .001

*Supplementary Table 4: Results of ANOVA identifying the effects of emotion type, face age, task difficulty and group (young vs. old) on RTs of happiness and anger emotion tasks.*

|  | *df* | *F* | *η²* | *p* |
| --- | --- | --- | --- | --- |
| Emotion Type | 1 | .008 | .000 | .927 |
| Emotion Type × Group | 1 | .073 | .003 | .789 |
| Error (Emotion Type) | 29 |  |  |  |
| Face Age | 1 | 1.637 | .053 | .211 |
| Face Age × Group | 1 | .000 | .000 | .987 |
| Error (Face Age) | 29 |  |  |  |
| Task Difficulty | 1 | 12.308 | .298 | .001* |
| Task Difficulty Group | 1 | 1.210 | .040 | .280 |
| Error (Task Difficulty) | 29 |  |  |  |
| Emotion Type × Face Age | 1 | 3.531 | .109 | .070 |
| Emotion Type × Face Age × Group | 1 | 1.586 | .052 | .218 |
| Error (Emotion Type × Face Age) | 29 |  |  |  |
| Emotion Type × Task Difficulty | 1 | .328 | .011 | .571 |
| Emotion Type × Task Difficulty × Group | 1 | .276 | .009 | .604 |
| Error (Emotion Type × Task Difficulty) | 29 |  |  |  |
| Face Age × Task Difficulty | 1 | 4.944 | .146 | .034* |
| Face Age × Task Difficulty × Group | 1 | 1.932 | .062 | .175 |
| Error (Face Age × Task Difficulty) | 29 |  |  |  |
| Emotion Type × Face Age × Task Difficulty | 1 | .732 | .025 | .399 |
| Emotion Type × Face Age × Task Difficulty × Group | 1 | .068 | .002 | .797 |
| Error (Emotion Type × Face Age × Task Difficulty) | 29 |  |  |  |
| Group | 1 | 1.940 | .063 | .174 |
| Error | 29 |  |  |  |

* *p* < .05, *** *p* < .001

**ERPs**

*Supplementary Table 5: Results of ANOVA identifying the effects of emotion type, face age, task difficulty and group (young vs. old) on cluster one ERPs during happiness and anger emotion tasks.*

|  | *df* | *F* | *η²* | *p* |
| --- | --- | --- | --- | --- |
| Emotion Type | 1 | .404 | .015 | .530 |
| Emotion Type × Group | 1 | .292 | .011 | .594 |
| Error (Emotion Type) | 26 |  |  |  |
| Face Age | 1 | .120 | .005 | .732 |
| Face Age × Group | 1 | 5.775 | .182 | .024* |
| Error (Face Age) | 26 |  |  |  |
| Task Difficulty | 1 | 4.974 | .161 | .035* |
| Task Difficulty × Group | 1 | 2.345 | .083 | .138 |
| Error (Task Difficulty) | 26 |  |  |  |
| Emotion Type × Face Age | 1 | .071 | .003 | .791 |
| Emotion Type × Face Age × Group | 1 | 2.084 | .074 | .161 |
| Error (Emotion Type × Face Age) | 26 |  |  |  |
| Emotion Type × Task Difficulty | 1 | 6.532 | .201 | .017* |
| Emotion Type × Task Difficulty × Group | 1 | .338 | .013 | .566 |
| Error (Emotion Type × Task Difficulty) | 26 |  |  |  |
| Face Age × Task Difficulty | 1 | .712 | .027 | .407 |
| Face Age × Task Difficulty × Group | 1 | 1.022 | .038 | .321 |
| Error (Face Age × Task Difficulty) | 26 |  |  |  |
| Emotion Type × Face Age × Task Difficulty | 1 | .092 | .004 | .764 |
| Emotion Type × Face Age × Task Difficulty × Group | 1 | .185 | .007 | .671 |
| Error (Emotion Type × Face Age × Task Difficulty) | 26 |  |  |  |
| Group | 1 | 20.273 | .438 | < .001*** |
| Error | 26 |  |  |  |

* *p* < .05, *** *p* < .001

*Supplementary Table 6: Results of ANOVA identifying the effects of emotion type, face age, task difficulty and group (young vs. old) on cluster two ERPs during happiness and anger emotion tasks.*

|  | *df* | *F* | *η²* | *Sig.* |
| --- | --- | --- | --- | --- |
| Emotion Type | 1 | 1.393 | .051 | .249 |
| Emotion Type × Group | 1 | 2.299 | .081 | .142 |
| Error (Emotion Type) | 26 |  |  |  |
| Face Age | 1 | .254 | .010 | .618 |
| Face Age × Group | 1 | 5.468 | .174 | .027* |
| Error (Face Age) | 26 |  |  |  |
| Task Difficulty | 1 | 6.813 | .208 | .015* |
| Task Difficulty Group | 1 | .513 | .019 | .480 |
| Error (Task Difficulty) | 26 |  |  |  |
| Emotion Type × Face Age | 1 | 1.252 | .046 | .273 |
| Emotion Type × Face Age × Group | 1 | 1.934 | .069 | .176 |
| Error (Emotion Type × Face Age) | 26 |  |  |  |
| Emotion Type × Task Difficulty | 1 | 2.619 | .092 | .118 |
| Emotion Type × Task Difficulty × Group | 1 | 5.781 | .182 | .024* |
| Error (Emotion Type × Task Difficulty) | 26 |  |  |  |
| Face Age × Task Difficulty | 1 | .810 | .030 | .376 |
| Face Age × Task Difficulty × Group | 1 | 1.126 | .042 | .298 |
| Error (Face Age × Task Difficulty) | 26 |  |  |  |
| Emotion Type × Face Age × Task Difficulty | 1 | .007 | .000 | .934 |
| Emotion Type × Face Age × Task Difficulty × Group | 1 | .032 | .001 | .859 |
| Error (Emotion Type × Face Age × Task Difficulty) | 26 |  |  |  |
| Group | 1 | 17.051 | .396 | < .001*** |
| Error | 26 |  |  |  |

* *p* < .05, *** *p* < .001
