# Supplementary material for: Investigating Age-Related Neural Compensation During Emotion Perception Using Electroencephalography
Source: Brain Sci. 2020 Jan 23;10(2):61. doi: 10.3390/brainsci10020061 (PMC7071462; doi:10.3390/brainsci10020061)
Supplement: Supplementary file 1 [file brainsci-10-00061-s001.zip › supplementary files/Supplementary 3_P100 and P300.docx]

**Group differences on P100 and P300 ERP components**

1. P100. According to prior work, responses to facial emotions may be seen as early as 100 ms [1,2]. This P100 represents an reflexive response processing of emotion with higher amplitude responses to fearful face presentation, compared to neutral facial emotion [3]. The P100 component (measured at the O1 and O2 electrode sites) is amygdalar-modulated visual perceptual response with emotional faces [4].

The results of current study revealed that older participants showed significantly higher early frontal and centromedial ERP positivity (100-200ms) across all facial emotions types including neutral emotion. This component seem to occur similar time to P100 (reflexive response to emotional faces), therefore older adults’ higher early frontal and centromedial ERP positivity (100-200ms) may indicate an extra input of cognitive resources along with the reflexive responses to emotional faces (P100) during facial processing.

Regarding the effect of age on ERP component P100, Pollock et al.’s (2012) reported that younger adults showed significantly higher P100 (time window: 70-170ms, electrodes O1 and O2) amplitudes for high-intensity angry faces than for high-intensity happy faces, but older adults showed almost identical P1 amplitudes for both high-intensity angry and happy faces. They interpreted that older adults have deficits in amygdala processing of facial emotion. To investigate the effect of group age on P100, we analysed young and older participants’ P100 (time window: 70-170ms, electrodes O1 and O2) using a mixed 2x2x2 ANOVA with emotions (anger, happiness), task difficulty (easy, hard) as within-participants factors, and group (young, old) as a between-participants factor. The results showed that the main effect of emotion was significant, *F*(1, 26) =10.337, *p* = .003. P100 was larger for happy faces than for angry faces. However, no other significant effects or interactions were found. Unlike Pollock et al.’s (2012), we did not found group difference on P100.


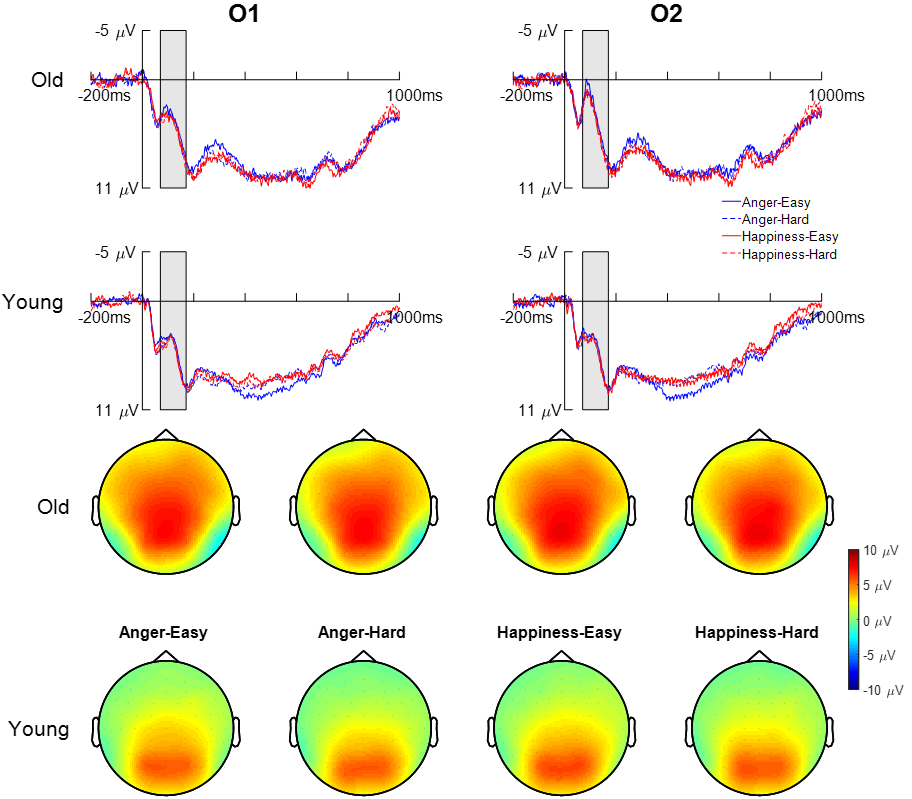


Figure 1. Grand average event-related brain potentials (ERPs) for P100 elicited by the experimental tasks as a function of emotions (anger, happiness), task difficulty (easy, hard), and group (young, old) for younger adults and older participants averaged across the electrodes O1 and O2. The unfilled rectangular boxes indicate the time window used to assess P1 (70–170 ms after stimulus onset). Negative is plotted upward and time zero represents stimulus onset. The scalp topography of the ERPs was obtained during the time window 70–170 ms after stimulus onset.

1. P300. To investigate the effect of group age on P300 at Fz, Cz and Pz, we analysed young and older participants’ P300 (electrodes Fz, Cz and Pz, separately) using three independent mixed 2x2x2 ANOVAs with emotions (anger, happiness), task difficulty (easy, hard) as within-participants factors, and group age (young, old) as a between-participants factor. We observed that P300 (300-450ms) of Fz was larger for older compared to younger participants [main effect of age group: *F*(1, 26) =8.332, *p* = 0.007]. However, no group difference were found on Cz and Pz. This finding further confirmed that the later positivity are more focused around frontal area.

**References**

1. Pizzagalli, D.; Regard, M.; Lehmann, D. Rapid emotional face processing in the human right and left brain hemispheres: an ERP study. *Neuroreport* **1999**, 10, 2691-2698.
2. Eimer, M.; Holmes, A. An ERP study on the time course of emotional face processing. *Neuroreport* **2002**, 13, 427-431.
3. Pollock, J.W.; Khoja, N.; Kaut, K.P.; Lien, M.C.; Allen, P. Electrophysiological evidence for adult age-related sparing and decrements in emotion perception and attention. *Frontiers in integrative neuroscience* **2012**, *6*, 60.
4. Rotshtein, P.; Richardson, M.P.; Winston, J.S.; Kiebel, S.J.; Vuilleumier, P.; Eimer, M.; Driver, J.; Dolan, R.J. Amygdala damage affects event‐related potentials for fearful faces at specific time windows. *Human brain mapping* **2010**, *31*(7), 1089-1105.
