# Supplementary material for: Investigating Age-Related Neural Compensation During Emotion Perception Using Electroencephalography
Source: Brain Sci. 2020 Jan 23;10(2):61. doi: 10.3390/brainsci10020061 (PMC7071462; doi:10.3390/brainsci10020061)
Supplement: Supplementary file 1 [file brainsci-10-00061-s001.zip › supplementary files/Supplementary 1_other results.docx]

***Reaction Times (RTs)***

*Perception of neutral facial expression*

No significant group difference was found for the RTs on neutral perception trials, and there was no other significant main effects or interaction (Figure 1).

*Perception of anger and happiness facial expressions*

A 2 (emotion type) × 2 (task difficulty) × 2 (face age) × 2 (group) mixed ANOVA on the RTs of anger and happiness perception tasks revealed significant main effect of difficulty [*F (1, 29) = 12.308, p = .001, η^2^ = .298*], which was due to participants performing significantly faster in easy tasks compared to hard tasks (Figure 1). Main effects of group, emotion type and face age were not significant.

The interaction of face age and task difficulty was significant, which was due to the RT of easy and hard conditions being significantly different in older face stimuli condition (*p < .001, d = 1.490*), but not in younger face stimuli condition.

**
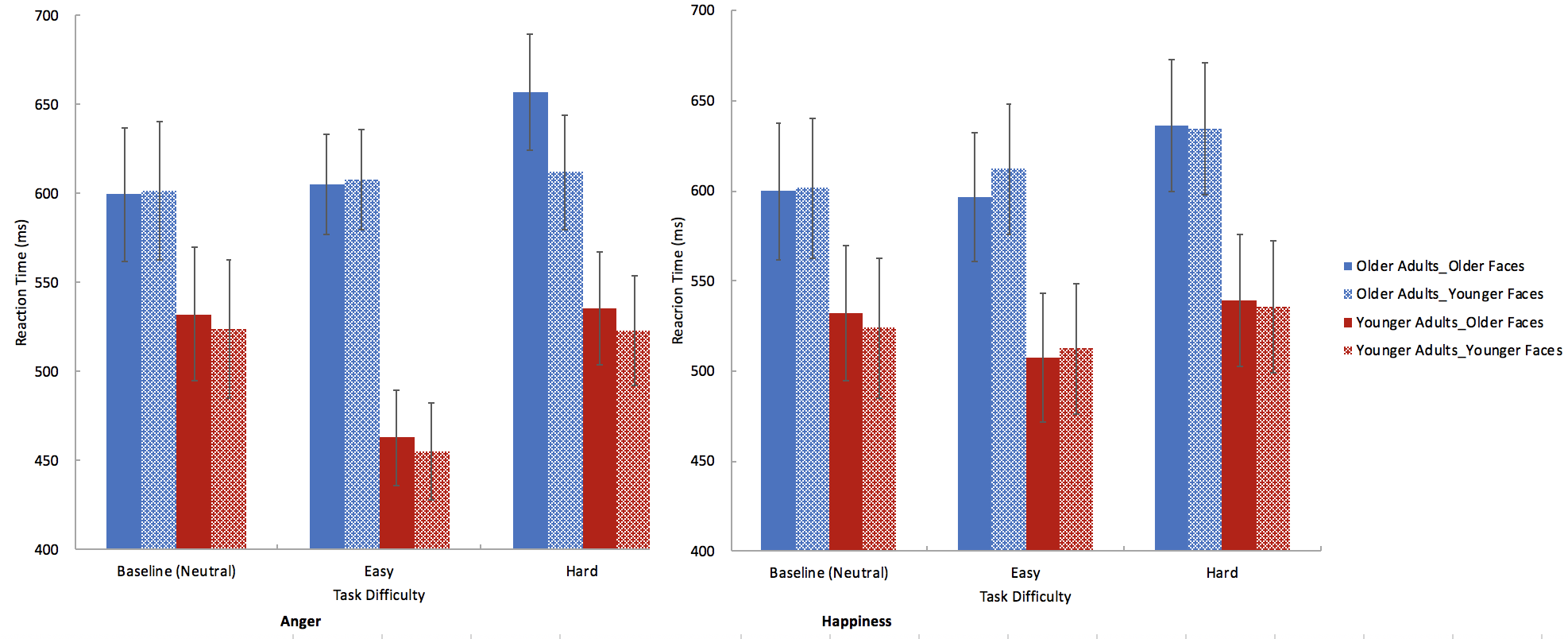
**

Figure 1. Older (bars in blue) and younger (bars in red) participants’ RT in anger (left graph) and happiness (right graph) experimental trials at different task difficulties [neutral (baseline), easy and hard]. Solid bars represent trials with older face stimuli, patterned bars represent trials with younger face stimuli. Error bars represents S.E.

**ERP results**

*Two groups’ cluster one mean ERP waveforms (100-200ms) during anger (a) and happiness (b) perceptual tasks.*


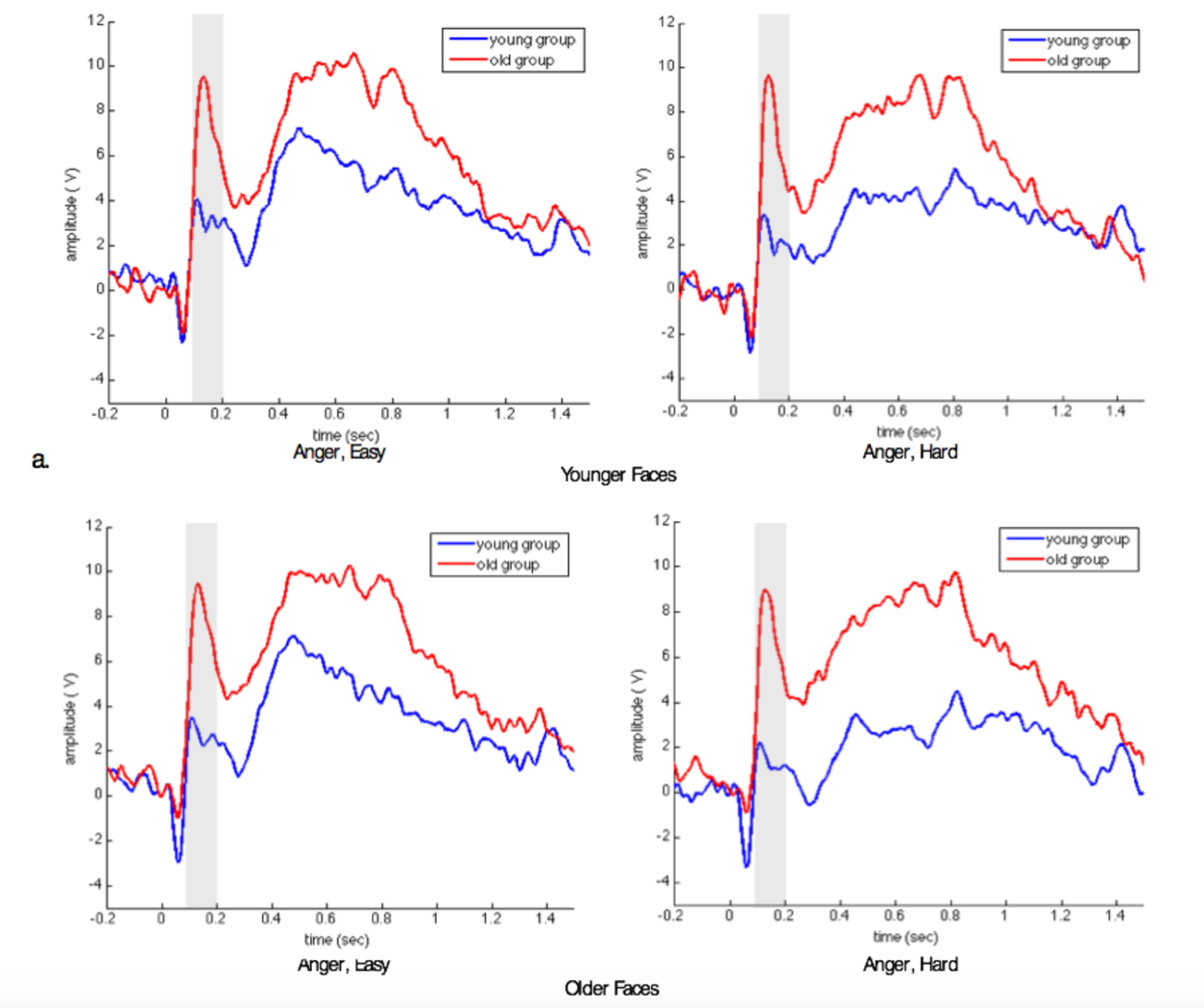


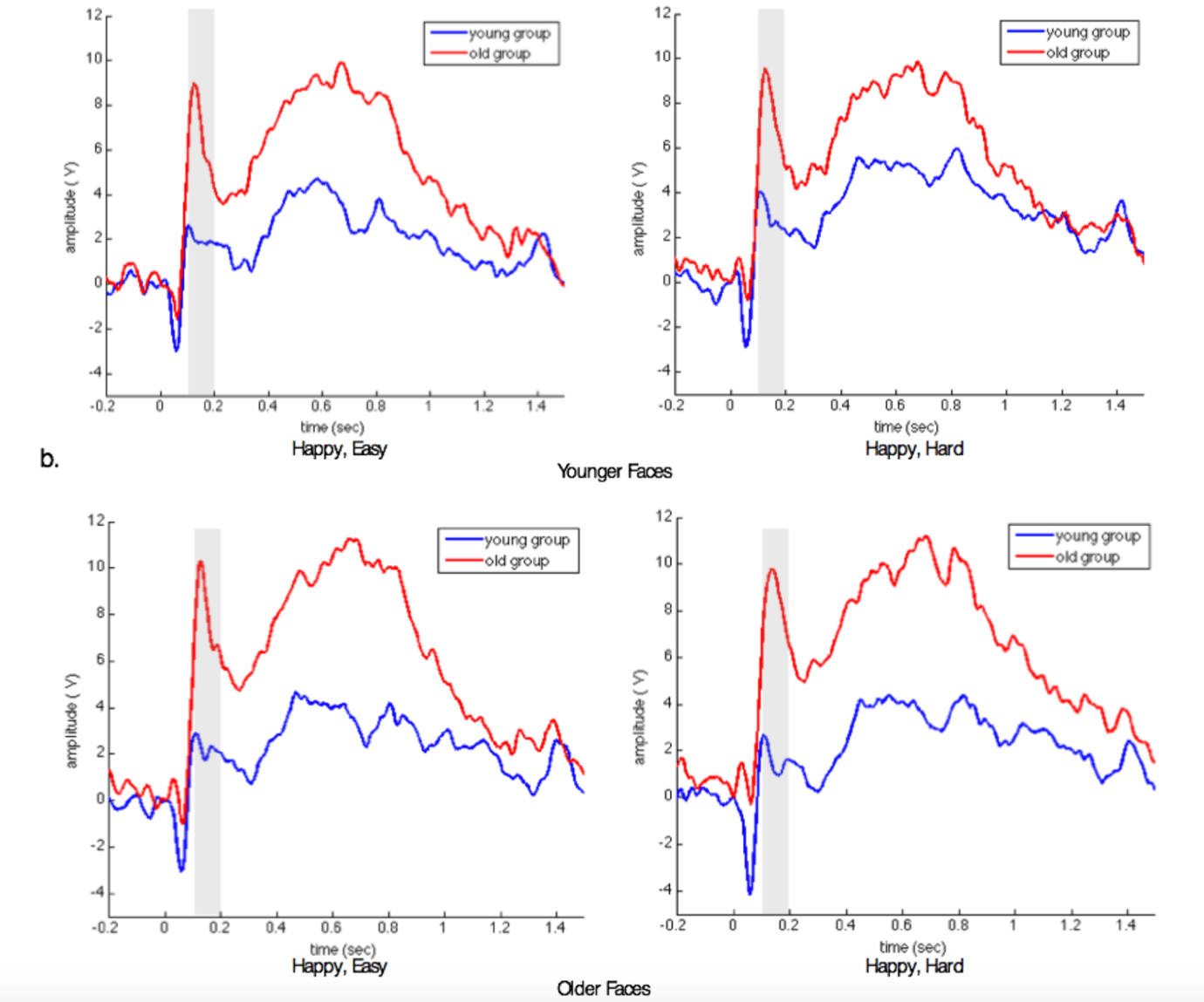


Figure 2. Younger (lines in blue) and older (lines in red) participants’ cluster one mean ERP waveforms (100-200ms) during anger (a) and happiness (b) perceptual tasks. The time window (100-200ms) used for subsequent statistical analysis are highlighted in grey.

*Two groups’ cluster two mean ERP waveforms (250-900ms) during anger (a) and happiness (b) perceptual tasks.*


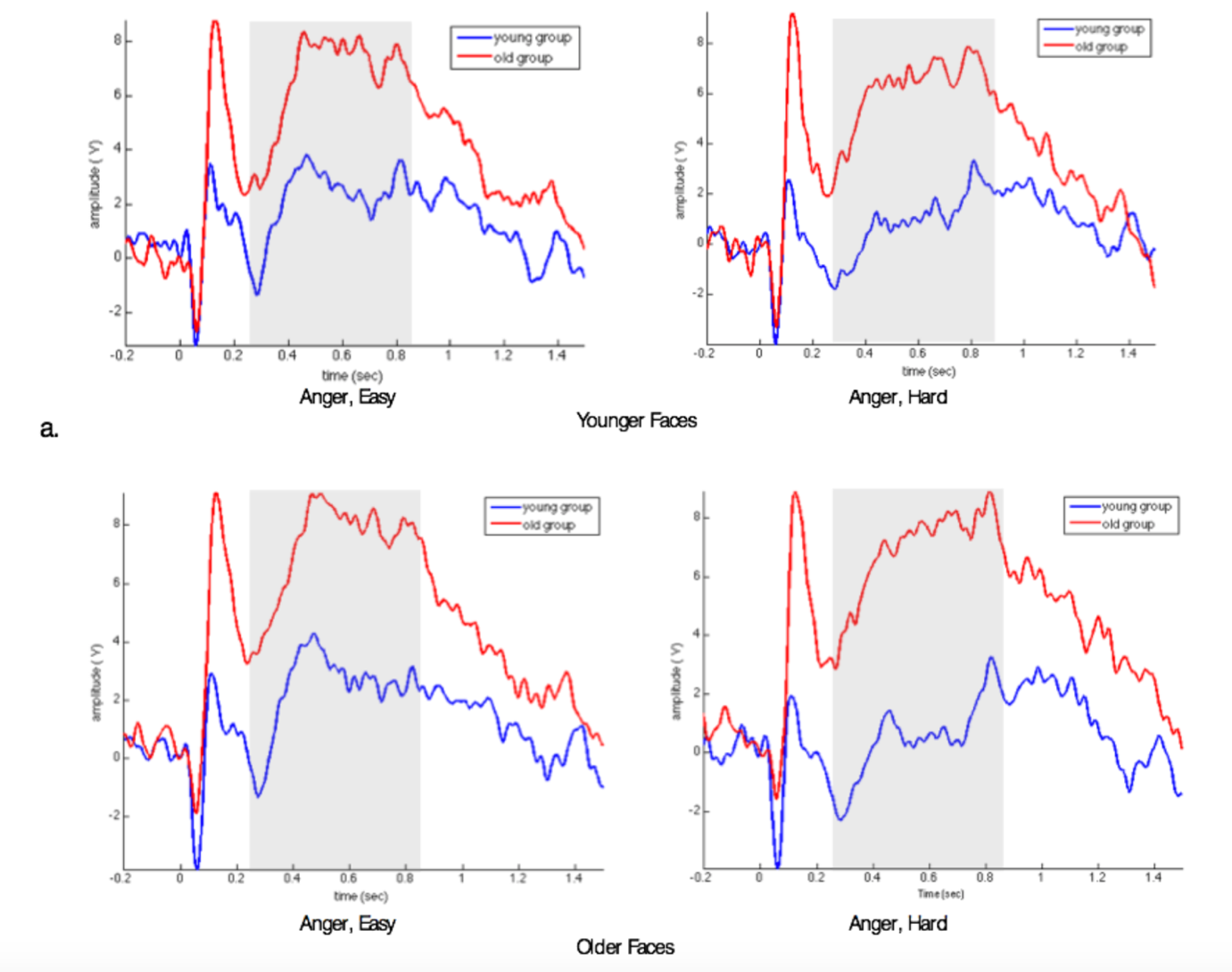


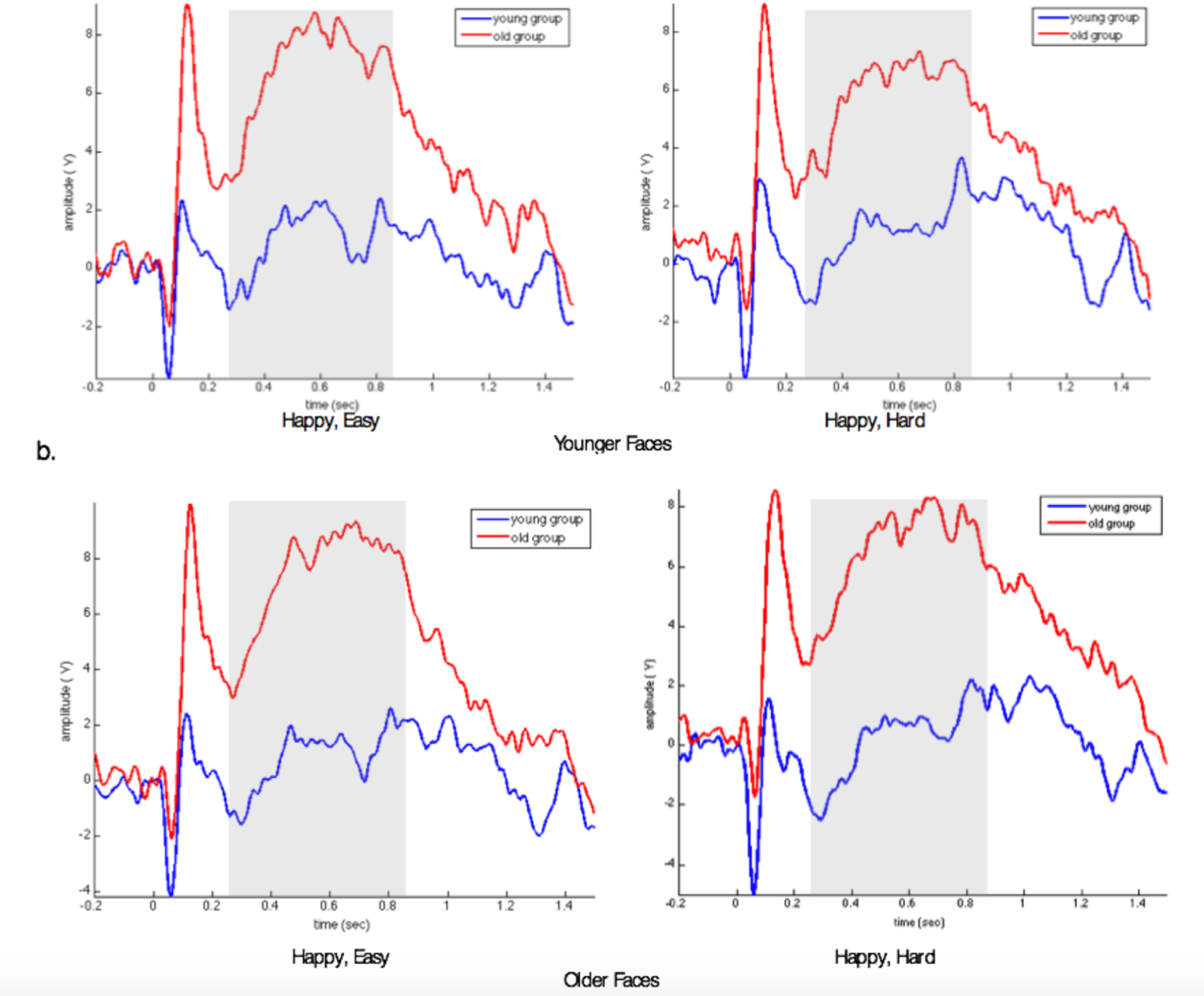


Figure 3. Younger (lines in blue) and older (lines in red) participants’ mean cluster two ERP waveforms (250-900ms) during anger (a) and happiness (b) perceptual tasks. The time window (250-900ms) used for subsequent statistical analysis are highlighted in grey.
